# Characteristics of patients with extracranial cervical artery dissections involving more than a single vessels: A subgroup analysis of STOP-CAD

**DOI:** 10.1093/esj/23969873251383313

**Published:** 2026-01-01

**Authors:** Issa Metanis, Favour Akpokiere, Liqi Shu, Yoel Schwartzmann, Hamza Jubran, Kateryna Antonenko, Mirjam R Heldner, Sara Rosa, Mafalda Delgado Soares, Stefan T Engelter, Josefin E Kaufmann, Christopher Kenan Traenka, Joao Pedro Marto, Michele Romoli, Adeel Zubair, Setareh Salehi Omran, Tamer Jubeh, Fatma Shalabi, Zafer Keser, Muhib Khan, Diana Aguiar DeSousa, Shadi Yaghi, Ronen R Leker

**Affiliations:** Department of Neurology, Hadassah-Hebrew University Medical Center, Jerusalem, Israel; Department of Neurology, Brown University Medical Center, Providence, RI, USA; Department of Neurology, Brown University Medical Center, Providence, RI, USA; Department of Neurology, Hadassah-Hebrew University Medical Center, Jerusalem, Israel; Department of Neurology, Hadassah-Hebrew University Medical Center, Jerusalem, Israel; Department of Neurology, Inselspital University Hospital, Bern, Switzerland; Department of Neurology, Inselspital University Hospital, Bern, Switzerland; Department of Neurology and Neuro-radiology, Centro Hospitalar Universitário Lisboa Central, Lisbon, Portugal; Department of Neurology and Neuro-radiology, Centro Hospitalar Universitário Lisboa Central, Lisbon, Portugal; Department Rehabilitation and Neurology, University Department of Geriatric Medicine FELIX PLATTER, University of Basel, Basel, Switzerland; Neurology and Stroke Center, University Hospital Basel, Depart. of Clinical Research, University Basel, Basel, Switzerland; Department Rehabilitation and Neurology, University Department of Geriatric Medicine FELIX PLATTER, University of Basel, Basel, Switzerland; Neurology and Stroke Center, University Hospital Basel, Depart. of Clinical Research, University Basel, Basel, Switzerland; Department Rehabilitation and Neurology, University Department of Geriatric Medicine FELIX PLATTER, University of Basel, Basel, Switzerland; Neurology and Stroke Center, University Hospital Basel, Depart. of Clinical Research, University Basel, Basel, Switzerland; Department of Neurology, Hospital de Egas Moniz, Centro Hospitalar Lisboa Ocidental, Lisbon, Portugal; Department of Neurosciences, Bufalini Hospital, Cesena, Italy; Department of Neurology, Yale University School of Medicine, New Haven, CT, USA; Department of Neurology, University of Colorado School of Medicine, Aurora, CO, USA; Department of Neurology, Hadassah-Hebrew University Medical Center, Jerusalem, Israel; Department of Neurology, Hadassah-Hebrew University Medical Center, Jerusalem, Israel; Department of Neurology, Mayo Clinic, Rochester, MN, USA; Department of Neurology, Mayo Clinic, Rochester, MN, USA; Michigan State University, Grand Rapids, MI, USA; Department of Neurology and Neuro-radiology, Centro Hospitalar Universitário Lisboa Central, Lisbon, Portugal; Department of Neurology, Brown University Medical Center, Providence, RI, USA; Department of Neurology, Hadassah-Hebrew University Medical Center, Jerusalem, Israel

**Keywords:** Dissection, vessel, single, multiple, extracranial

## Abstract

**Introduction:**

Cervical arterial dissections (CeAD) can involve either single (sCeAD) or multiple (mCeAD) arteries. Whether the involvement of a single versus multiple arteries is associated with outcomes remains unclear. We aimed to study associations between the number of affected arteries and clinical, imaging and outcome parameters.

**Patients and methods:**

Patients with CeAD from the STOP-CAD multicenter registry study were included. Clinical, imaging, treatment and outcome parameters were compared between patients with sCeAD and mCeAD. Regression analyses were performed to identify associations with multi-arteries involvement.

**Results:**

Overall, 3858 STOP-CAD patients were included in this analysis and 443 (11.5%) had mCeAD. The presence of mCeAD was associated with age (adjusted odds ratio [aOR] 95% confidence intervals [95% CI] 0.99; (0.98–1.00)), female sex (aOR 1.5; 95% CI 1.17–1.91), recent upper respiratory infection (aOR 2.25; 95% CI 1.55–3.27), presence of connective tissue disease (aOR 3.11; 95% CI 2.32–4.17), severe arterial stenosis (aOR 1.95; 95% CI 1.95–2.58), intracranial extension (aOR 1.47; 95% CI 1.04–2.09), vertebral artery involvement (aOR 2.50; 95% CI 1.94–3.22) and presence of dissecting aneurysm (aOR 2.59; 95% CI 1.95–3.42). In adjusted analyses, mCeAD was not associated with clinical outcomes (ischemic stroke, mortality, and sICH; all *p* > 0.05).

**Conclusions:**

mCeAD does not appear to increase risk of subsequent stroke as compared to sCAD despite baseline risk factors suggestive of vasculopathy. mCeAD patients who did develop a stroke presented with milder strokes and less often had vessel occlusions compared to those with sCeAD. The presence of mCeAD did not impact outcomes.

## Graphical Abstract

**Graphical Abstract:**
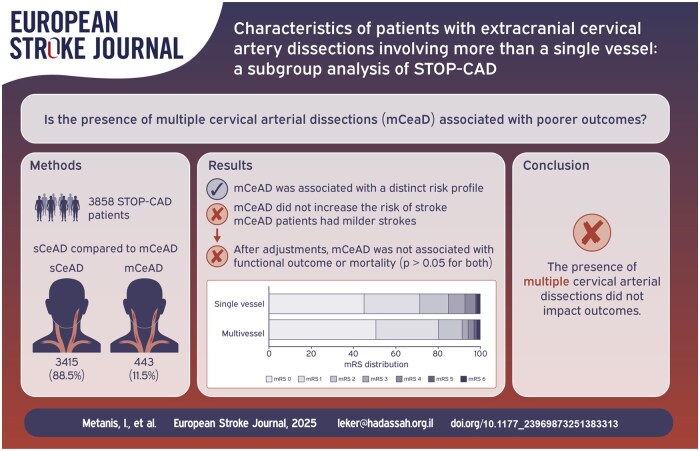

## Introduction

Cervical arterial dissections (CeAD) are a common cause of stroke in young adults and can lead to significant morbidity.^1–5^ CeAD can involve only a single vessel (sCeAD) or concomitantly involve multiple arteries (mCeAD).^6,7^ Previous studies found mCeAD to represent ~15% of all CeAD and patients with mCeAD had higher frequencies of incident strokes.^6,7^ However, whether sCeAD and mCeAD differ in clinical, imaging or outcome parameters remains largely unexplored. Furthermore, the risk factors associated with developing mCeAD were not sufficiently explored and it remains possible that concomitant multi-vessel involvement could put patients at risk of infarction and may thus lead to poorer outcomes. Moreover, patients with mCeAD were found to have impaired vasoreactivity and this could also translate to more severe stroke due to impaired collateral flow.^8^ Therefore, we aimed to analyze data from sCeAD and mCeAD patients included in a large multicenter international registry to identify risk factors associated with mCeAD and to explore whether multivessel involvement affects clinical outcomes.

## Patients and methods

This was a retrospective analysis of patients with CeAD included in the STOP-CAD observational international multicenter registry study. The study was approved by the institutional ethics committee of all participating centers following approval of the Institutional Review Board at Lifespan (1894800-5) granting waiver from obtaining informed consent due to the retrospective nature of the study.

The methods and results of the main study were published before.^9^ Briefly, the diagnosis of CeAD was confirmed on imaging studies demonstrating extracranial intimal flaps, double lumens, flame-shaped occlusions, progressive vessel narrowing or tapering of the vessel lumen leading to distal occlusion. All included patients also had a clinical suspicion of dissection. Patients with or without stroke were included and the only inclusion criteria was the presence of CeAD on imaging. Patients with chronic incidental findings of dissection were excluded, as were those with traumatic dissections, as defined elsewhere.^10,11^ Patients presenting with focal neurological symptoms and signs lasting more than 24 h or acute focal brain lesions compatible with stroke on imaging, were diagnosed with stroke. In contrast, patients without acute or delayed (i.e. within a few days) imaging confirmation of stroke (e.g. patients presenting with isolated cranial nerve palsies, isolated tinnitus or Horner’s syndrome) were classified as non-stroke patients.

**Figure 1.**
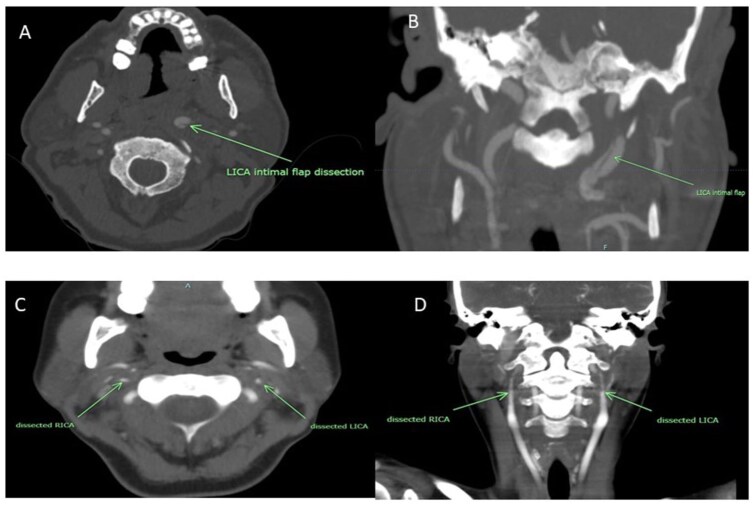
CT angiography images from patients with single (a and b) and multiple (c and d) vessel dissections in axial (a and c) and coronal (b and d) plains. Green arrows point to the dissections. LICA: left internal carotid artery; RICA: right internal carotid artery.

In this analysis, patients were categorized based on whether they had involvement of only a single vessel (sCeAD) or more than one vessel (mCeAD). mCeAD was defined as evidence of concomitant acute dissections affecting non-continuous vessels (i.e. continuous dissections involving the common carotid and internal carotid artery on the same side were classified as sCeAD, whereas dissections involving bilateral internal carotid or vertebral arteries, or those both the internal carotid and vertebral arteries, were classified as mCeAD, Figure 1). Patients with prior dissections and a new dissection involving only one vessel were classified as sCeAD. Patients without any extradural dissection were excluded from this analysis.

Data were collected as previously described in the original STOP CAD publication.^9^ Briefly, data were extracted form electronic medical records, including outpatient visits to neurology and neurosurgery clinics, and included demographics, stroke and dissection-related risk factors, clinical stroke characteristics and severity, and imaging findings. Vessel stenosis was categorized as mild (<50%), moderate (50%–70%), severe (70%–99%) or complete occlusion. We also recorded the presence of intracranial extension. The frequency of imaging biomarkers of dissection including intimal flaps, double lumen, tapering, narrowing, and occlusion was also documented. Connective tissue disorders was defined as the presence of clinically diagnosed connective tissue disorder such as Marfan’s disease, Ehrler Danlos syndrome, or any other known connective tissue disorder. Fibromuscular dysplasia (FMD) can lead to collagen turnover abnormalities^12^ and its presence was separately documented.

Information on treatments administered and outcome parameters was also collected. The primary outcome for the current analysis was favorable functional outcome, defined as modified Rankin Scale score (mRS) of ⩽2 at 90 days post CeAD. Secondary outcome parameters included the presence of any intracranial hemorrhage (ICH), symptomatic ICH (sICH), and the risk of developing mCeAD. In addition, data on survival, recurrent stroke and recurrent dissection rates at preset time points of 90 and 180 days post admission and the time of the last follow-up visit were documented.

## Statistical analysis

Continuous variables are reported as medians with interquartile ranges (IQRs) or as means and standard deviations (SDs) and categorical variables as counts and percentages. Baseline demographic, clinical and imaging characteristics were compared between the single-vessel (sCeAD) and multi-vessel (mCeAD) groups. Categorical variables were compared using Pearson’s chi-square test, while continuous and ordinal variables were analyzed using the Wilcoxon rank-sum test.

In order to identify individual variables that were significant predictors of our prespecified outcomes we performed a stepwise logistic regression including all variables that yielded a *p* value of <0.1 for each of the outcome measures including functional outcome, mortality, stroke during follow up and sICH and then adjusted for these for each outcome. For each outcome, the logistic regression models were fitted with multi-vessel involvement versus single-vessel involvement as the exposure and fully adjusted for variables that yielded a *p* < 0.1 in the unadjusted analysis. In addition, the distribution of 90-day mRS outcomes, stratified by single-versus multi-vessel involvement, was illustrated using a Grotta-style stacked horizontal bar chart, depicting the proportion of patients in each mRS category at 90 days.

To identify predictors of multi-vessel dissection, each candidate variable was first evaluated in univariable logistic regression. All significant predictors were then entered simultaneously into a multivariable logistic regression model to determine independent associations with multi-vessel involvement.

All data management and statistical analyses were conducted in Stata (version 18.0; StataCorp, College Station, TX). Two-sided *p*-values < 0.05 were considered statistically significant.

## Results

Of the 4023 patients included in the STOP-CAD study 165 (4%) had evidence of isolated intracranial dissections and were excluded from the current analysis. Overall, 3858 patients with CeAD remained in the analysis, with a median age of 46 years (IQR 37–56) and a median admission NIHSS of 1 (IQR 0–7) for the entire group. Overall, 44% of the patients were female.

Of the included patients, 443 (11.5%) were diagnosed with mCeAD (Supplemental Figure 1) and 60% of those were female (Table 1). Compared to patients with sCeAD, those with mCeAD were significantly younger (median, IQR; 42 [34–52] vs 47 [38–56]; *p* < 0.0001) and significantly more likely to have a history of migraine (23% vs 16%, *p* < 0.0001). They also more frequently reported a recent upper respiratory infection (11% vs 6%; *p* < 0.0001) and mild neck trauma (27% vs 22%; *p* = 0.016), but less often had classical stroke risk factors such as diabetes and hyperlipidemia (Table 1). Patients in the mCeAD group more commonly had a history of connective tissue disease (22% vs 8%; *p* < 0.0001). Fibromuscular dysplasia (FMD) was also more prevalent in mCeAD patients (21% vs 7%; *p* < 0.001). Patients in the mCeAD group presented less often with stroke symptoms and signs (54% vs 66%; *p* < 0.0001) and with less severe neurological deficits (NIHSS median [IQR], 1 [0–4] vs. 1 [0–7]; *p* = 0.0005). Vertebral artery involvement was more common in mCeAD patients (65% vs 42%; *p* < 0.0001), whereas carotid artery involvement was similar between the two groups. Vessel occlusions were less frequent in this group (29% vs 38%; *p* < 0.0001), as were acute infarcts on initial imaging (52% vs 58%; *p* = 0.012). Intracranial extension of the dissection was more frequently observed in the mCeAD group (Table 1).

**Table 1.** Baseline characteristics and treatment modalities.

Group (number)	All (*n* = 3858)	Single vessel (*n* = 3415)	Multi-vessel (*n* = 443)	*p*
Median age (interquartile range)	46 (37–56)	47 (38–56)	42 (34–52)	<0.0001
Mean age and standard deviation	47.3 (13.2)	47.8 (13.2)	43.3 (12.4)	<0.0001
Female sex	1733 (44.9%)	1465 (42.9%)	268 (60.5%)	<0.0001
Race				
White	2849 (73.9%)	2505 (73.4%)	344 (77.7%)	
Black	230 (6.0%)	210 (6.2%)	20 (4.5%)	
Asian	127 (3.3%)	110 (3.2%)	17 (3.8%)	
Other	652 (16.9%)	590 (17.3%)	62 (14.0%)	0.128
Pre stroke mRS (median, IQR)	0 (0–0)	0 (0–0)	0 (0–0)	0.364
Prior disability				
None (mRS 0)	3513 (92.2%)	3104 (92.0%)	409 (93.6%)	
Mild (mRS 1–2)	254 (6.7%)	228 (6.8%)	26 (5.9%)	
Moderate (mRS 3)	29 (0.8%)	29 (0.9%)	0 (0%)	
Severe (mRS 4–5)	14 (0.4%)	12 (0.4%)	2 (0.5%)	0.226
Risk factors				
Migraine	650 (16.9%)	549 (16.1%)	101 (22.8%)	<0.0001
Hypertension	1348 (34.9%)	1211 (35.5%)	137 (30.9%)	0.060
Diabetes	324 (8.4%)	308 (9.0%)	16 (3.6%)	<0.0001
Hyperlipidemia	877 (22.7%)	807 (23.6%)	70 (15.8%)	<0.0001
Active smoking	741 (19.2%)	671 (19.7%)	70 (15.8%)	0.053
Recent upper respiratory infection	247 (6.4%)	199 (5.8%)	48 (10.8%)	<0.0001
Neck trauma	863 (22.4%)	744 (21.8%)	119 (26.9%)	0.016
Connective tissue disease	81 (2.1%)	63 (1.8%)	18 (4.1%)	0.002
Fibromuscular dysplasia	320 (8.3)	229 (6.7)	91 (20.6)	<0.0001
Presenting symptoms				
Stroke	2496 (64.7%)	2255 (66.1%)	241 (54.4%)	
TIA	464 (12.0%)	399 (11.7%)	65 (14.7%)	
No stroke/TIA	896 (23.2%)	759 (22.2%)	137 (30.9%)	<0.0001
NIHSS on admission (Median (IQR))	1 (0–7)	1 (0–7)	1 (0–4)	0.0005
Acute infarct on imaging	2232 (57.9%)	2000 (58.6%)	232 (52.4%)	0.013
Baseline hemorrhagic conversion	166 (4.3%)	154 (4.5%)	12 (2.7%)	0.079
% Vessel stenosis				
Mild (less than 50%) to Moderate (50%–70%)	1390 (36.4%)	1237 (36.6%)	153 (34.6%)	
Severe (70%–99%)	1029 (26.9%)	868 (25.7%)	161 (36.4%)	
Occlusion (100%)	1401 (36.7%)	1273 (37.7%)	128 (29.0%)	<0.0001
Intracranial extension	341 (8.9%)	282 (8.3%)	59 (13.9%)	<0.0001
Carotid dissection	2235 (57.9%)	1983 (58.1%)	252 (56.9%)	0.635
Vertebral dissection	1721 (44.6%)	1432 (41.9%)	289 (65.2%)	<0.0001
Intravenous thrombolysis	620 (16.1%)	571 (16.7%)	49 (11.1%)	0.002
Endovascular stent and thrombectomy	451 (11.7%)	403 (11.8%)	48 (10.8%)	0.552
Initial Heparin/LMWH use	983 (25.5%)	848 (24.8%)	135 (30.5%)	0.010
DOAC treatment	439 (11.4%)	386 (11.3%)	53 (12.0%)	0.680
Vitamin K antagonist treatment	537 (13.9%)	465 (13.6%)	72 (16.3%)	0.131
Initial anti-platelets	3518 (91.2%)	3115 (91.2%)	403 (91.0%)	0.864

IQR: interquartile range; mRS: modified Rankin Scale; TIA: transient ischemic attack.

Predictors of developing mCeAD were studied next, adjusting for baseline variables (Table 2). The presence of mCeAD was significantly associated with age (adjusted odds ratio [aOR]; 95% confidence intervals [95% CI] 0.99; (0.98–1.00), female sex (aOR 1.5; 95% CI 1.17–1.91), recent upper respiratory infection (aOR 2.25; 95% CI 1.55–3.27) and presence of connective tissue disease (aOR 3.11; 95% CI 2.32–4.17). Imaging parameters related to development of mCeAD included severe vessel stenosis (aOR 1.95; 95% CI 1.48–2.58), presence of intimal flaps (aOR 1.68; 95% CI 1.25–2.26), intracranial extension of dissections (aOR 1.47; 95% CI 1.04–2.09), vertebral artery involvement (aOR 2.50; 95% CI 1.95–3.42) and presence of pseudoaneurysms (aOR 2.59; 95% CI 1.95–3.42).

**Table 2.** Factors associated with multi vessel dissections.

Predictor	Adjusted^a^ OR (95% CI)	*p*
Age	0.99 (0.98–1.00)	0.005
Female sex	1.50 (1.17–1.91)	0.001
Migraine	0.94 (0.71–1.26)	0.696
Diabetes	0.60 (0.34–1.05)	0.075
Hyperlipidemia	0.81 (0.58–1.12)	0.203
Upper respiratory infection	2.25 (1.55–3.27)	<0.001
Neck trauma	1.07 (0.82–1.38)	0.631
Connective tissue disorder	3.11 (2.32–4.17)	<0.001
Ischemic presentation	0.92 (0.76–1.11)	0.381
NIHSS at admission	1.00 (0.98–1.02)	0.969
Infarct on imaging	0.96 (0.74–1.24)	0.762
Vessel stenosis: Severe (vs Mild/Mod)	1.95 (1.48–2.58)	<0.001
Vessel stenosis: Occlusion (vs Mild/Mod)	1.46 (1.07–1.99)	0.016
Intracranial extension	1.47 (1.04–2.09)	0.030
Vertebral dissection	2.50 (1.94–3.22)	<0.001
Pseudo aneurysm	2.59 (1.95–3.42)	<0.001

NIHSS: National Institutes of Health Stroke Scale.

^a^Adjusted refers to multivariable regression including all variables to determine independent determinants.

Patients with mCeAD were treated less often with systemic thrombolytics (11% vs 17%; *p* = 0.002) but the rates of stent placement did not differ between the groups (Table 3). Patients in the mCeAD group were more frequently started on low molecular weight heparin, whereas oral antithrombotic treatment did not differ between groups (Table 3).

**Table 3.** Outcomes in patients with single or multiple vessel dissections.

Variable	All (*n*= 3,858)	Single vessel (*n*=3,415)	Multi vessel (*n*=443)	*p*
NIHSS day 1(Median (IQR))	1 (0–7)	1 (0–7)	1 (0–4)	0.0005
mRS discharge (Median (IQR))	1 (0–2)	1 (0–2)	1 (0–2)	0.0030
Disability on discharge:				
None (mRS 0)	1212 (33.4%)	1060 (32.9%)	152 (36.7%)	
Mild (mRS 1-2)	1611 (44.4%)	1418 (44.1%)	193 (46.6%)	
Moderate (mRS 3)	320 (8.8%)	290 (9.0%)	30 (7.2%)	
Severe (mRS 4-5)	489 (13.5%)	450 (14.0%)	39 (9.4%)	0.028
mRS day 90 (Median (IQR)	1 (0–2)	1 (0–2)	0 (0–1)	
	*n*=3,092	*n*=2,723	*n*=369	0.0023
Disability at day 90:				
None (mRS 0)	1403 (45.4%)	1217 (44.7%)	186 (50.4%)	
Mild (mRS 1-2)	1245 (40.3%)	1094 (40.2%)	151 (40.9%)	
Moderate (mRS 3)	218 (7.1%)	208 (7.6%)	10 (2.7%)	
Severe (mRS 4-5)	162 (5.2%)	147 (5.4%)	15 (4.1%)	0.002

IQR: interquartile range; LMWH: low molecular weight heparin; mRS: modified Rankin Scale.

**Figure 2.**
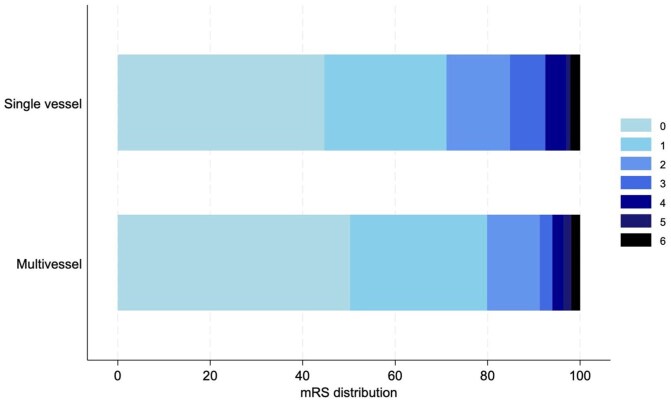
Bar graph showing the distribution of outcomes according to modified Rankin score at 90 days post presentation.

Outcomes data at discharge were available for 3632/3858 (94%) and for 3028/3858 (78%) at 90 days post-presentation (2666/3415 (78%) sCeAD and 362/443 (82%) mCeAD patients). In unadjusted analyses, both median disability scores and shifts in disability showed statistically significant better outcomes in the mCeAD group at both time points (Figure 2). Rates of symptomatic intracranial hemorrhage, recurrent dissections and recurrent stroke were very low and did not differ between the groups.

After appropriate adjustment for each individual outcome, the rates of favorable outcome did not significantly differ between the groups (Table 4). Similarly, mortality rates at 180 days post-presentation, as well as rates of sICH or stroke did not differ between the groups (Table 4).

**Table 4.** Multi-vessel dissection as predictor for outcomes in adjusted analysis.

Outcome	Adjusted OR (95% CI)	*p-*Value	Adjusted for
Favorable outcome at 90 days (mRS 0–2)	0.98 (0.61–1.58)	0.942	Age at diagnosis, sex, migraine, diabetes, hyperlipidemia, recent upper respiratory infection, neck trauma, presenting ischemia, NIHSS at admission, acute infarct on imaging, vessel stenosis severity, vertebral dissection, connective tissue disease, FMD
Death by 180 days	2.33 (0.93–5.82)	0.070	Age at diagnosis, sex, migraine, diabetes, presenting ischemia, NIHSS at admission, acute infarct on imaging, vessel stenosis severity
Symptomatic ICH	1.33 (0.46–3.85)	0.600	Age at diagnosis, sex, NIHSS at admission, acute infarct on imaging, vessel stenosis severity, vertebral dissection
Stroke during follow-up at 90 days	1.27 (0.82–1.96)	0.281	Hyperlipidemia, neck trauma, presenting ischemia, acute infarct on imaging, vessel stenosis severity

FMD: fibromuscular dysplasia; ICH: intracerebral hemorrhage; mRS: modified Rankin Scale; NIHSS: National Institutes of Health Stroke Scale.

## Discussion

The main findings of the current analysis are that mCeAD are not infrequent, are more commonly seen in females and that despite some differences in the prevalence of risk factors between mCeAD and sCeAD patients, the risk of stroke is not higher in patients with mCeAD. Furthermore, patients with mCeAD appeared to have less severe strokes, were less frequently treated with intravenous thrombolytics and showed more favorable outcomes compared to those with sCeAD in unadjusted analyses. However, after adjustment for confounding factors, there were no significant differences in any outcome parameters or complications between mCeAD and sCeAD patients.

Several potential mechanisms could contribute to differences in pathophysiology and outcomes between sCeAD and mCeAD. For example, FMD and connective tissue disorders such as Marfan’s disease and Ehlers-Danlos syndrome which could lead to a higher frequency of mCeAD and theoretically impact outcomes, may be more common in patients with mCeAD.^13–17^ FMD was indeed identified in the current study as one of the most common cause of dissections and the presence of FMD or connective tissue disorders remained a strong predictor for developing mCeAD. This is particularly important because those patients may at a higher risk of future dissections and stroke. Furthermore, genetic factors also play a role in CeAD and specific genetic variations may influence the likelihood of CeAD occurrence and recurrence as well as affect recovery.^16,18–20^ However, these genetic variants were not studied in the current study and we cannot ascertain their possible link to the development of mCeAD. Patients with mCeAD were found to have impaired vasomotor reactivity to isosorbide dinitrate in both the carotid and vertebral artery, which may limit collateral flow and lead to more extensive damage in dissections causing stroke.^8^ However, we could not replicate those findings and patients with mCeAD actually had milder strokes and better outcomes in the current analysis.

In STOP-CAD, which is one of the largest studies of patients with CeAD, mCeAD was observed in 11.5% of included patients. This rate is slightly lower than those reported in previous studies, which may be due to the more stringent diagnostic criteria for mCeAD applied in our study.^6,7^

Known risk factors for dissection, such as migraine, recent upper respiratory infection and neck trauma, were more frequent among patients with mCeAD, while other traditional stroke risk factors, including diabetes and hyperlipidemia, were less frequent in mCeAD patients. Some of these associations were also reported in a previous smaller study,^6^ whereas other factors, such as migraine and the absence of diabetes or hyperlipidemia, have not been previously described as influencing the risk of developing mCeAD. In the current analysis, patients with sCeAD were older than those with mCeAD, which may partly explain the higher prevalence of traditional stroke risk factors in the sCeAD group and could also account for the higher rates of unfavorable outcomes observed in sCeAD patients.

Surprisingly, patients in the mCeAD group had a lower frequency of clinical stroke as well as stroke on imaging, despite the involvement of multiple vascular territories and a higher frequency of intracranial extension of the dissection. This finding could suggest that only some of the lesions were symptomatic, while some were not. Alternatively, it is possible that asymptomatic dissections were detected in the pre-symptomatic state and that rapid, appropriate treatment given upon detection of the symptomatic lesion may have prevented stroke in arterial territories that were not yet ischemic. However, because we did not systematically collect data on lesion distribution or volume on imaging, nor or on the co-existence of multiple stroke syndromes occurring simultaneously, we cannot confirm or refute this hypothesis.

There were several differences in clinical presentation and vessel involved between the groups. For example, vertebral dissections were more common and ischemic strokes were less common in the mCeAD group. This could result from the fact that the sensitivity of imaging studies for posterior circulation strokes is less than 100%.^21^ Interestingly, systemic thrombolysis was more commonly used in patients with sCeAD, who also appeared to have more frequent complete vessel occlusions and more severe strokes, likely explaining these treatment decisions. The presence of vessel occlusions may also have been responsible for the higher rates of poor outcomes observed in these patients. After adjustments, the chances of attaining favorable outcomes at discharge and at 90 days post-admission were similar in the mCeAD and sCeAD patients, despite the higher rates of thrombolytics given to patients with sCeAD, which may suggest that some patients with sCeAD did not recanalise with intravenous thrombolysis. The observation of similar outcomes in the two groups may be related to the lower initial stroke severity and the lower frequency of complete vessel occlusions in the mCeAD group, both of which are known to be associated with less favorable outcomes.^4,10^ This may also be explained by the higher likelihood of receiving initial treatment with low molecular weight heparin anticoagulants, which has shown a trend toward better outcomes in the STOP-CAD trial and in a recent meta-analysis.^9,22^

The current study has several limitations. First, the retrospective registry design might be source of several potential biases, including selection bias, reporting bias and bias by indication. Second, imaging and clinical data were adjudicated locally at each participating center and we did not have a central imaging core lab. This could have led to overdiagnosis of mCeAD in some cases. However, our rates of mCeAD detection were actually lower than previously reported, suggesting this was unlikely. Furthermore, because chronic dissection may be difficult to distinguish from acute dissections on imaging, we cannot exclude the possibility that some of the dissections observed in the mCeAD group represented chronic dissections separated in time from the acute symptomatic dissection. However, chronic dissections (clinical and/or on imaging) were deemed an exclusion criteria in the current study, which should have limited the presence of such misclassifications. Another limitation pertains to the lack of information regarding the number of concurrent clinical stroke syndromes in individual patients with multi-vessel dissections, as well as the number, volume, and distribution of ischemic lesions on imaging.

The current study has several strengths including an all-inclusive design enrolling all patients with spontaneous dissections and its international multicenter design, which enhances the generalizability of the results.

In conclusion, the current findings suggest that patients with concomitant multi-vessel dissection exhibit distinct clinical features indicative of a generalized inherited or acquired vasculopathy. However, concomitant involvement of multiple vessels implicated in the CeAD process is not associated with differences in clinical outcomes in patients with CeAD.

## Supplementary Material

ds-eso_23969873251383313

## Data sharing

Study data can be made available on reasonable request to the principal investigator. The requests must be accompanied by detailed study proposals, a description of study objectives and a valid analysis plan. Additional material might be requested during the assessments of the study eligibility for data sharing, which must be approved by the principal investigators of the study. Each request will be examined for compatibility with regulatory requirements.
