# Correction to: Characteristics of patients with extracranial cervical artery dissections involving more than a single vessels: A subgroup analysis of STOP-CAD

**DOI:** 10.1093/esj/aakag017

**Published:** 2026-02-23

This is a correction to: Issa Metanis, Favour Akpokiere, Liqi Shu, Yoel Schwartzmann, Hamza Jubran, Kateryna Antonenko, Mirjam R Heldner, Sara Rosa, Mafalda Delgado Soares, Stefan T Engelter, Josefin E Kaufmann, Christopher Kenan Traenka, Joao Pedro Marto, Michele Romoli, Adeel Zubair, Setareh Salehi Omran, Tamer Jubeh, Fatma Shalabi, Zafer Keser, Muhib Khan, Diana Aguiar DeSousa, Shadi Yaghi, Ronen R Leker, Characteristics of patients with extracranial cervical artery dissections involving more than a single vessels: A subgroup analysis of STOP-CAD, *European Stroke Journal*, Volume 11, Issue 1, January 2026, 23969873251383313, https://doi.org/10.1093/esj/23969873251383313

The last name of a co-author has been emended to read: “Zafer Keser”.

