# Supplementary figures and images for: Characteristics of patients with extracranial cervical artery dissections involving more than a single vessels: A subgroup analysis of STOP-CAD
Source: Eur Stroke J. 2026 Jan 1;11(1):23969873251383313. doi: 10.1093/esj/23969873251383313 (PMC12866210; doi:10.1093/esj/23969873251383313)

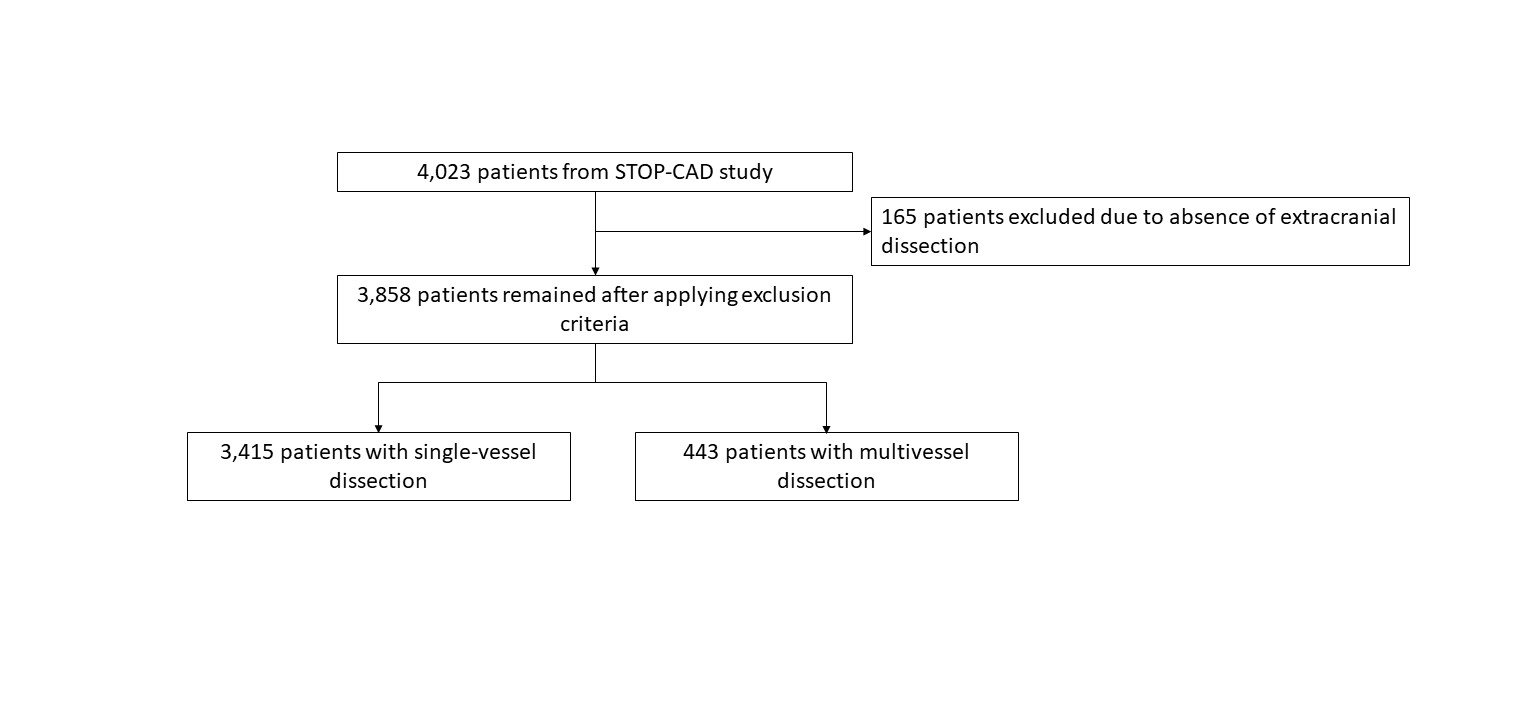

Supplement: ds-eso_23969873251383313 [file ds-eso_23969873251383313.zip › sj-jpg-1-eso-10.1177_23969873251383313.jpg]
